# Linking megathrust earthquakes to brittle deformation in a fossil accretionary complex

**DOI:** 10.1038/ncomms8504

**Published:** 2015-06-24

**Authors:** Armin Dielforder, Hauke Vollstaedt, Torsten Vennemann, Alfons Berger, Marco Herwegh

**Affiliations:** 1 Institute of Geological Sciences, University of Bern, Baltzerstrasse 1+3, Bern CH-3012, Switzerland; 2 Center for Space and Habitability, University of Bern, Sidlerstrasse 5, Bern CH-3012, Switzerland; 3 Institute of Earth Surface Dynamics, University of Lausanne, Gêopolis 4634, Lausanne CH-1015, Switzerland

## Abstract

Seismological data from recent subduction earthquakes suggest that megathrust earthquakes induce transient stress changes in the upper plate that shift accretionary wedges into an unstable state. These stress changes have, however, never been linked to geological structures preserved in fossil accretionary complexes. The importance of coseismically induced wedge failure has therefore remained largely elusive. Here we show that brittle faulting and vein formation in the palaeo-accretionary complex of the European Alps record stress changes generated by subduction-related earthquakes. Early veins formed at shallow levels by bedding-parallel shear during coseismic compression of the outer wedge. In contrast, subsequent vein formation occurred by normal faulting and extensional fracturing at deeper levels in response to coseismic extension of the inner wedge. Our study demonstrates how mineral veins can be used to reveal the dynamics of outer and inner wedges, which respond in opposite ways to megathrust earthquakes by compressional and extensional faulting, respectively.

## Abstract (web summary)

Recent megathrust earthquakes have been documented to cause large-scale stress changes, although this has not been identified in a fossil system. Here, the authors present data that establish a link between the observations at active subduction zones and the structural record preserved in ancient mountain belts.

## Graphical abstract

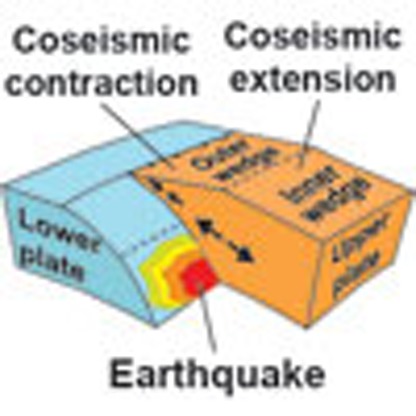

Accretionary prisms form along convergent margins when sediments are scraped off from the subducting plate and transferred to the upper plate. Sediments accreted near the toe of the wedge are commonly transported towards greater depths and experience diagenetic to low-grade metamorphic conditions before being exhumed to the surface^1^. Previous work on accretionary wedges^2^,^3^,^4^,^5^ and plate interfaces^6^,^7^,^8^,^9^ showed how mineral reactions, lithification and the development of a tectonic fabric affect the rheology of accreted sediments and control the onset of seismicity within the unstable zone of subduction megathrusts. The effect of subduction-zone or megathrust earthquakes on deformation in the overlying accretionary wedge, however, has remained largely unnoticed. New hints for a link between megathrust earthquakes and internal wedge deformation come from active convergent margins. Geodetic and seismological data recorded along the Chilean and Japanese forearcs documented a significant increase in upper-plate seismicity after the 2010 *M*_w_ 8.8 Maule and the 2011 *M*_w_ 9.0 Tohoku earthquakes, and revealed that coseismically induced stress changes activate splay and normal faults in the upper plate^10^,^11^,^12^. This suggests that megathrust earthquakes affect the mechanical state of the overlying wedge and are expected to leave a widespread signature in the seismic record of rocks.

Here we present a detailed field study that documents the long-term conditions of brittle rock failure in a domain of a fossil accretionary complex that evolved from an outer into an inner wedge, overlying the aseismic and seismogenic part of the basal detachment, respectively. Our combination of structural information with isotope data (Sr, C and O) and predictions of a Mohr–Coulomb wedge model enables us to better understand how accretionary wedges respond to megathrust earthquakes. We conclude that coseismically induced stress changes can trigger wedge internal deformation and leave a discernable signature in the seismic record of rocks.

## Results

### Geological background

**Figure 1:**
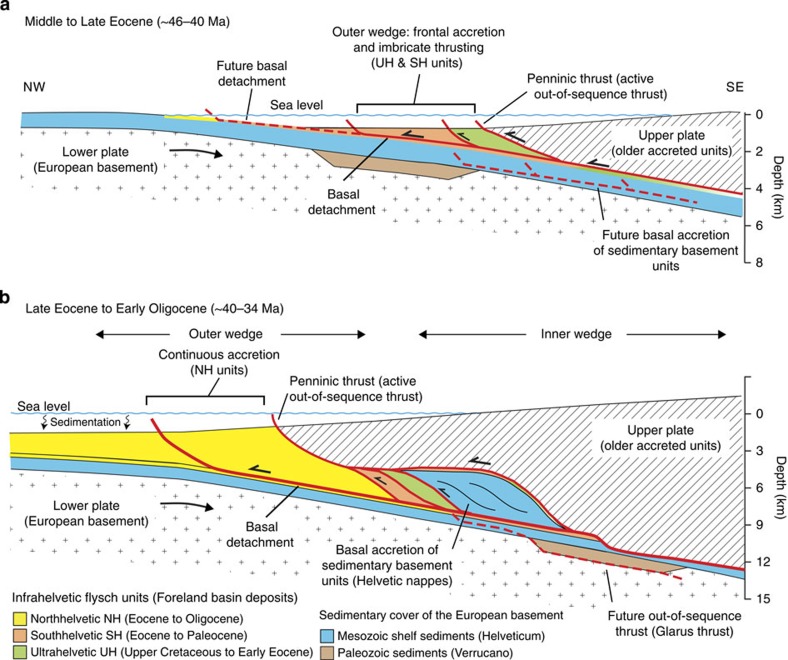
Schematic reconstruction of Paleogene Alpine wedge (**a**) Frontal accretion of the Ultrahelvetic (UH) and Southhelvetic (SH) units starts at ∼46 Ma. The basal detachment and imbricate thrust faults propagate into the direction of the foreland. (**b**) Around 40 Ma the Northhelvetic (NH) unit is accreted to the wedge. Continuous out-of-sequence thrusting along the Penninic thrust buries the nappe stack beneath the wedge. In the inner wedge the Mesozoic sedimentary basement units (Helvetic nappes) are accreted to the base of the wedge and thrusted on top of the Infrahelvetic flysch units. The dashed red line indicates the position of a future splay fault (Glarus thrust) that cuts at a later stage through the wedge. Age estimates for the onset of accretion from ref. 13. Depths are rough estimates, vertical exaggeration is ∼1.5–2.

The study area is located in the Palaeogene accretionary complex of the central European Alps, which is one of the best documented fossil systems worldwide. The high relief of ∼2,600 m provides unique insights into the internal architecture of this orogenic wedge. The area comprises an exhumed ∼4-km-thick sequence of hemipelagic shales, marls and limestones, as well as syn-orogenic turbidites (flysch) including volcanic detritus^13^,^14^,^15^. The sediments were deposited in an underfilled foreland basin^15^ and show a similar sedimentological composition as trench-fill sediments found along active accretionary margins. The sequence consists of three thrust slices of Upper Cretaceous to early Oligocene age, known as Infrahelvetic flysch units (IFUs). Frontal accretion of these thrust slices to the Alpine wedge commenced in the middle Eocene during the subduction of the passive European continental margin (Fig. 1a). The IFUs were subsequently transported towards greater depths due to continuous plate convergence and out-of-sequence thrusting within the rearward part of the wedge (Fig. 1b). The prograde evolution of the IFU ceased in the early Miocene^13^,^16^ when the coupling along the plate interface strengthened and the basal detachment stepped down into the European continental crust, causing underplating, uplift and exhumation of crystalline basement units and the IFUs^17^,^18^. Until then, ∼150 km of thinned continental crust had been subducted beneath the upper plate^15^. At present, the IFUs are sandwiched between the autochthonous European basement and overlying nappes that were transported along out-of-sequence thrusts on top of the IFUs since the Oligocene^13^. Peak metamorphic conditions reached 220–240 °C and ∼0.2 GPa in the northern and ∼320–350 °C and ∼0.3 GPa in the southern part of the study area^19^ (Supplementary Fig. 1).

### Field observations

**Figure 2:**
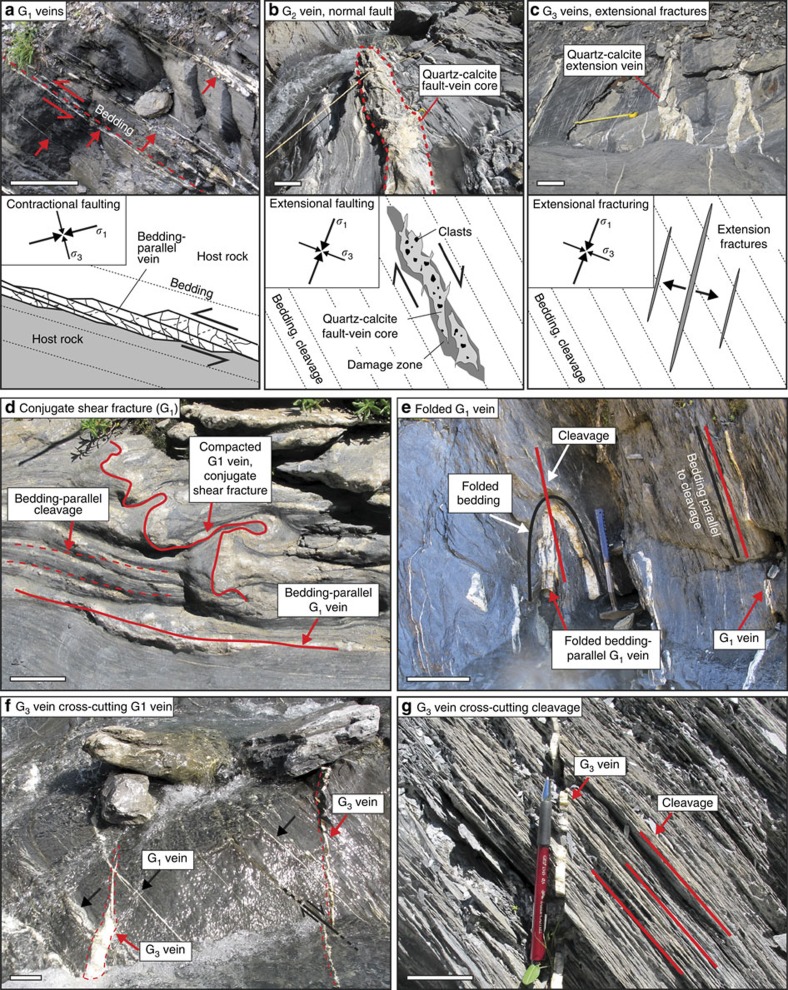
Examples of mineral veins and cross-cutting relationships. (**a**) G_1_ veins (red arrows) were formed by bedding-parallel slip and record contraction within the wedge. (**b**) G_2_ veins represent mineralized cores of steep normal faults, contain clasts of brecciated host rock and are surrounded by a damage zone. (**c**) G_3_ veins comprise of mineralized extension fractures. Both, G_2_- and G_3_ veins record pulses of extensional brittle deformation within the wedge. The orientation of the principal stresses *σ*_1_ and *σ*_3_ during vein formation is depicted in the upper left corner of the sketches. (**d**) Bedding-parallel G_1_ vein with conjugate shear fracture. The conjugate shear fracture was intensively shortened during sediment compaction. (**e**) G_1_ vein folded during interlayer folding. (**f**) Two G_3_ veins cross-cutting G_1_ veins. The G_3_ vein on the right is offset along a minor, not mineralized normal fault. (**g**) G_3_ veins cross-cutting cleavage. Scale bar, 25 cm (**a**–**c**,**e**,**f**). Scale bar, 5 cm (**d**,**g**).

Deformation related to accretion involved sediment compaction, folding, boudinage, dissolution-precipitation creep and faulting. Faults and fractures form mineral veins, indicating the presence of fluids during fracturing. The veins are present in all three thrust slices of the IFU and can be traced on the metre to decametre scale. Cross-cutting relationships indicate a successive formation of three groups of mineral veins (Fig. 2), which we used to decipher the history of brittle rock failure within the accretionary complex. The first group (G_1_) consists of calcite shear veins that are widespread in fine-grained lithologies, such as slates, marlstones and siltstones, but are absent in coarse-grained sandstones. The majority of G_1_ veins were formed incrementally along bedding planes during reverse faulting, documenting horizontal contraction within the wedge (Fig. 2a; Supplementary Fig. 2). Conjugate shear fractures are sometimes developed within marlstones at an angle of ∼60°±10° to bedding-parallel shear veins. Oblique shear veins are intensively affected by sediment compaction and shortened by ∼30–60% (Fig. 2d), suggesting a formation at a shallow level within the wedge. G_1_ veins and bedding planes were folded, which is most evident where interlayer folding occurs (Fig. 2e). Veins of the second and third group (G_2_ and G_3_) cross-cut G_1_ veins, folds and cleavage (Fig. 2f,g) and consist mainly of quartz and calcite with minor amounts of sulfides, plagioclase and white mica. They are less common than G_1_ veins and occur commonly in clusters, predominantly in the southern domain of the study area (Supplementary Fig. 1). G_2_ veins represent mineralized fault cores of steep normal faults and include centimetre-sized clasts of brecciated host rock (Fig. 2b). Similar to G_1_ veins, they are well developed within fine-grained lithologies. In contrast, G_3_ veins formed by steep-dipping extension fractures (Fig. 2c) are present in all lithologies. Together with G_2_ veins they record extensional brittle deformation within the wedge.

### Temperature and depth of vein formation

**Figure 3:**
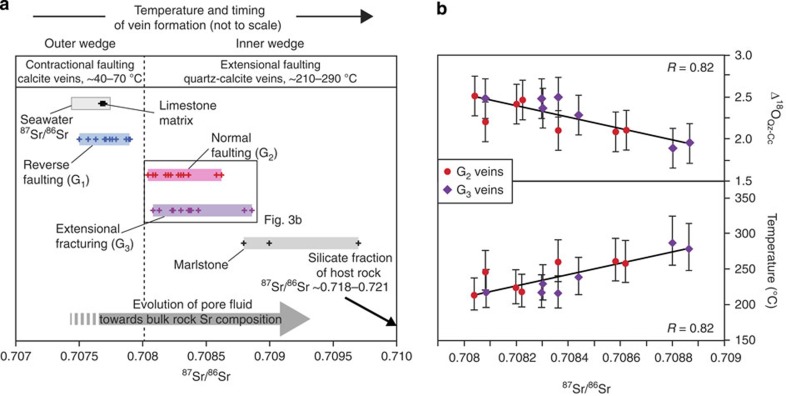
Sr and O isotope systematics. The ^87^Sr/^86^Sr ratios (*n*=41) and Δ^18^O_Qz−Cc_ values (*n*=14) of mineral veins record the development of the pore fluid during the prograde evolution of the wedge. (**a**) Calcite veins (G_1_, blue) show ^87^Sr/^86^Sr ratios similar to sea water and limestone matrix and document reverse faulting within shallow levels of the wedge during carbonate diagenesis (∼40–70 °C). Quartz-calcite veins (G_2_, red and G_3_, purple) show higher ^87^Sr/^86^Sr ratios, which develop towards the Sr signature of the host rock (marlstone). The veins indicate extensional faulting and fracturing within deeper levels of the wedge. 2σ uncertainties are smaller than the symbols. (**b**) The oxygen isotope fractionation data between quartz and calcite (Δ^18^O_Qz−Cc_) from G_2_- and G_3_ veins show a good correlation (*R*=0.82) with the ^87^Sr/^86^Sr ratios. The respective temperatures suggest a formation of G_2_- and G_3_ veins over similar time intervals during the prograde evolution of the wedge between ∼210 and 290 °C.

We studied the strontium (^87^Sr/^86^Sr), carbon (δ^13^C, ‰ VPDB) and oxygen (δ^18^O, ‰ VSMOW) isotope systematics of mineral veins and host rock to further constrain the setting and relative timing of vein formation during the prograde evolution of the Alpine wedge (Fig. 3; Supplementary Tables 1 and 2). Our approach is based on the following two assumptions: (1) during sedimentation the pore fluid is derived from sea water, which is not in isotopic equilibrium with the host rock. During the transport of sediments towards greater depths and associated diagenetic and low-grade metamorphic reactions, the Sr isotopic signature of the pore fluid will evolve towards that of the host rock. The ^87^Sr/^86^Sr ratios of mineral veins formed at different depths will therefore record the evolution of the pore fluid. (2) C and O isotopes bear information on carbonate diagenesis, fluid sources, open- vs closed-system behaviour and temperature-controlled isotope fractionation. All samples were taken from a homogenous, ∼300-m-thick rock unit (Globotruncana marl) to minimize potential variations in the isotopic composition of the host rock. The unit was deposited in a calcite sea (that is, marine conditions that favour the precipitation of low-Mg calcite, instead of aragonite and high-Mg calcite) and has a stratigraphic age of Santonian–Campanian^20^ (∼86−72 Ma). As the marl is devoid of dolomite and aragonite, in the following we focus on calcite.

#### G_1_ veins

Calcite of G_1_ veins show ^87^Sr/^86^Sr ratios of 0.7075–0.7079, which are similar to sea water at the time of sediment deposition^21^ (^87^Sr/^86^Sr ∼0.7074–0.7077 between 86 and 72 Ma) and to bulk carbonate leachate obtained from a limestone bed intercalated within the Globotruncana marl (^87^Sr/^86^Sr value of ∼0.7077). This documents the precipitation of G_1_-vein carbonates from a pore fluid that was dominated by a Sr isotopic signature of contemporaneous sea water. Such a signature is commonly found in pore fluids of marine sediments subjected to carbonate diagenesis, that is, the recrystallization of biogenic carbonates to secondary inorganic calcite^22^,^23^,^24^. Biogenic carbonates bear an identical ^87^Sr/^86^Sr ratio as contemporaneous sea water, but their Sr concentration is 1–3 orders of magnitude higher than in pore fluids or sea water^25^. The high Sr content of the calcite will effectively buffer the isotopic signature of the pore fluid during calcite recrystallization^22^,^23^,^24^. Hence, the overlap in the ^87^Sr/^86^Sr ratios of G_1_ veins, limestone beds and contemporaneous sea water suggests vein formation during carbonate diagenesis, whose conditions will be constrained in the following.

The δ^18^O values of calcite cement from host rock and adjacent calcite from G_1_ veins cluster tightly between 20.5 and 25.6‰ and 22.1 and 26.3‰, respectively (Supplementary Fig. 3), indicating that the O-isotopic composition of the fluid was buffered by calcite cements during vein formation. A similar picture emerges from the δ^13^C values of cements and vein calcite, which range from −0.6 to +2.1‰ and +1.5 to +2.3‰, respectively. These values lie well within the range of common Cretaceous limestones^26^. A positive correlation between δ^18^O and δ^13^C values reflects the successive cementation of the host rock and points to an equilibrium fractionation with increasing temperature towards lower values during diagenetic conditions^27^,^28^. Assuming a near-marine δ^18^O composition of the pore fluid during carbonate diagenesis^27^,^28^,^29^, the δ^18^O values of calcite cements indicate recrystallization at temperatures of ∼40–70 °C. These values agree with previous temperature estimates for carbonate diagenesis obtained from deep-sea drilling projects^23^,^24^ and a recent clumped-isotope study on cementation and matrix recrystallization, which yielded temperatures of ∼14–65 °C for calcite diagenesis^30^. Given the pristine and marine-equilibrated isotopic signature in our data set, we exclude any substantial post-diagenetic calcite recrystallization^28^ (for example, during low-grade metamorphism) and conclude that the formation of G_1_ veins took place over a temperature range of ∼40–70 °C. Assuming a geothermal gradient of 20–30 °C km^−1^, this corresponds to ∼1–4 km depth and suggests a formation in the shallow outer wedge.

#### G_2_- and G_3_ veins

Analyses of whole-rock samples and leached silicate fractions of the Globotruncana marl yield relatively high ^87^Sr/^86^Sr ratios of 0.7088–0.7097 and 0.7174–0.7207, respectively (Supplementary Table 3). The pore fluid is therefore expected to evolve towards higher ^87^Sr/^86^Sr ratios during progressive burial. In fact, carbonates of G_2_- and G_3_ veins show ^87^Sr/^86^Sr ratios of 0.7080–0.7089, which is distinctly higher than the ^87^Sr/^86^Sr of G_1_ veins (0.7077±0.0002). Similar to G_1_ veins, the δ^18^O and δ^13^C values of calcite from G_2_- and G_3_ veins lie between 22.2 and 23.2‰ and 1.5 and 2.2‰, respectively, indicating that also during later stages of vein formation the C and O-isotopic composition of the pore fluid was buffered by the host rock (Supplementary Fig. 3). This enables us to constrain the formation temperatures of G_2_- and G_3_ veins based on the oxygen isotope fractionation between quartz and calcite^31^,^32^ (Δ^18^O_Qz−Cc_=δ^18^O_Qz_−δ^18^O_Cc_), which grew coevally in equilibrium with the fluid (Supplementary Fig. 2). The δ^18^O values of quartz from G_2_- and G_3_ veins are systematically higher than in calcites and range from 24.1 to 25.5‰. The related Δ^18^O_Qz−Cc_ values for G_2_- and G_3_ veins range from 2.1 to 2.5‰ and 1.9 to 2.5‰, respectively, and yield formation temperatures of 210–260 °C and 220–290 °C, respectively (Fig. 3; Supplementary Table 1). The temperature range corresponds to a formation at ∼7–15 km depth and suggests an origin within deeper levels of the inner wedge. Furthermore, the Δ^18^O_Qz−Cc_ values and formation temperatures show a good correlation with the ^87^Sr/^86^Sr ratios of vein carbonates (*R*=0.82), which indicates the progressive interaction of the pore fluid with the host rock and the formation of G_2_- and G_3_ veins during the prograde evolution of the wedge.

Taken together, our combined structural and geochemical data reveal two distinct modes of brittle deformation within the Alpine wedge: (1) contractional faulting within the outer wedge, and (2) extensional faulting and fracturing within the inner wedge.

### Dynamic Mohr–Coulomb wedge

**Figure 4:**
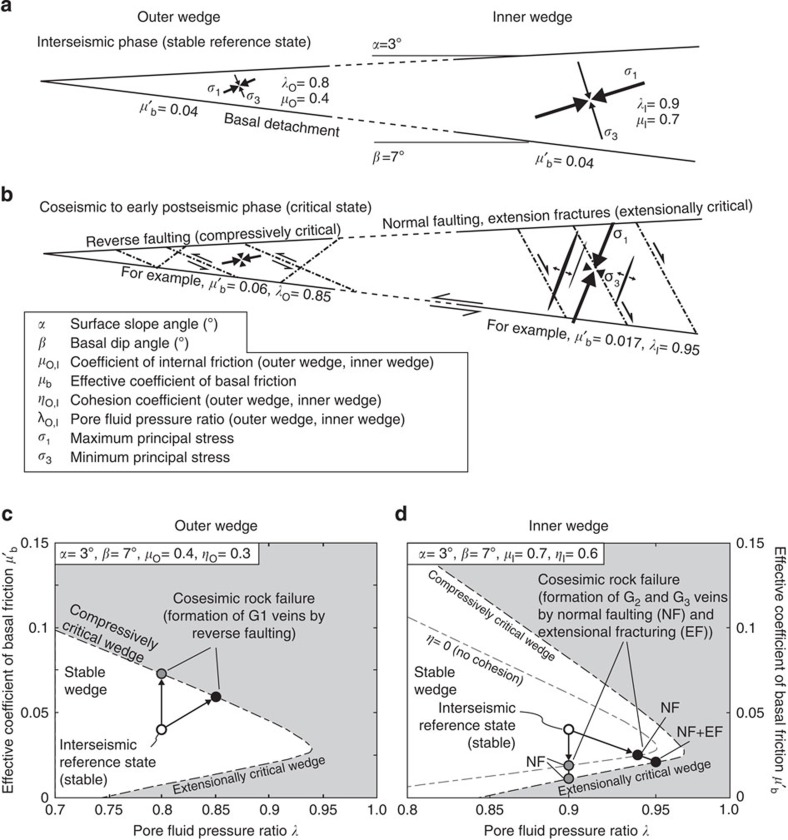
Dynamic Mohr–Coulomb wedge analysis. (**a**) Interseismic reference state showing model parameters and calculated stress fields for the outer and inner wedge. During the interseismic period the whole wedge is in a stable state. (**b**) Outer and inner wedge during the coseismic to early postseismic phase. The outer wedge is in a compressively critical state and the inner wedge in an extensionally critical state. (**c**,**d**) Stability field diagrams showing the critical values of *μ*'_b_ as a function of *λ* for the outer and inner wedge, respectively. The interseismic reference state is indicated by the open circle in the stable field. Coseismic strengthening (**c**) or weakening (**d**) shifts the wedge towards the unstable state. Exemplary solutions for failure in the outer and inner wedge are indicated by the grey and black circles. The grey circles represent failure due to strengthening or weakening of the basal detachment solely, whereas the black circles show solutions considering a coseismic increase in the pore fluid pressure also.

To investigate how the two modes of brittle deformation are related to the large-scale tectonic setting at convergent margins, we apply the dynamic Mohr–Coulomb wedge theory^33^ to the Alpine prism. This theory expands the original Coulomb wedge model by considering stress changes during the subduction earthquake cycle, and predicts both the orientation of the maximum principal stress (*σ*_1_) and the stress conditions under which the wedge fails. This enables us to constrain the timing of vein formation within the subduction earthquake cycle. The model is constrained by the wedge geometry, the strength of the wedge and the basal detachment, as well as the pore fluid pressure ratio *λ* (where *λ* is the ratio of pore fluid pressure and lithostatic pressure). The parameters were estimated from previous wedge analyses^33^,^34^ and balanced cross-sections^35^ and are given in Fig. 4. We further explored the parameter space within the range of reasonable values (Supplementary Fig. 4). The model wedge is divided into an outer and an inner wedge (Fig. 4a; Supplementary Table 4). The inner wedge overlies the unstable, seismogenic zone of the basal detachment where earthquake nucleation occurs. This part of the plate boundary is characterized by velocity-weakening behaviour, which is modelled by a coseismic decrease in basal friction (*μ′*_b_). The outer wedge overlies the (conditionally) stable zone of the basal detachment, which shows velocity-strengthening behaviour and is modelled by a coseismic increase in *μ′*_b_. The division of the basal detachment into a velocity-strengthening and a velocity-weakening part assumes a common behaviour for the subduction fault. Exceptional situations, in which the earthquake rupture propagates through the upper aseismic zone, suggesting that the shallow part of the detachment experience only little strengthening or even weakening^36^, are not further considered here.

The dynamic Mohr–Coulomb wedge analysis reveals that both parts of the wedge are stable during the interseismic phase (Fig. 4a), and rock failure could only occur by an increase in pore fluid pressure within the wedge to near-lithostatic values (*λ*>0.92 and >0.96 for the outer and inner wedge, respectively). For the outer wedge, we consider this as unlikely, as such high fluid pressures are not supported by seismological and borehole data^37^,^38^. In the inner wedge, near-lithostatic fluid pressures persisting during the interseismic period would result in the formation of subhorizontal hydrofractures, which we do not observe in the field. Strong earthquakes, however, can induce stress variations, which will affect the mechanical state of the entire wedge. Coseismic strengthening of the basal detachment leads to a compression of the outer wedge, pushing it towards the compressively critical state. In this process, *σ*_1_ rotates slightly towards the horizontal (Fig. 4b). The outer wedge fails when the basal friction *μ′*_b_ increases to ∼0.07, which results in the formation of thrust faults (Fig. 4c). Taking a coseismic increase in fluid pressure into account^33^ (for example, from *λ*=0.8 to *λ*=0.85), rock failure occurs at an even lower increase of the basal friction. The inner wedge behaves in a converse manner to the outer wedge. Coseismic weakening of the basal detachment brings the wedge towards an extensionally critical state, and *σ*_1_ rotates towards the vertical (Fig. 4b). Wedge instability occurs at fluid pressures of *λ*∼0.90–0.95 and associated drops in *μ′*_b_ of 0.02–0.03 (Fig. 4d). Depending on the cohesional strength of the rocks, the inner wedge fails either by normal faulting (for example, along weak planes such as cleavage) or extensional fracturing (Fig. 4d; Supplementary Fig. 5). In summary, our wedge analysis indicates that instabilities occur during transient stress changes, which are induced by megathrust earthquakes and persist over the co- to early postseismic periods (see also ref. 33). Moreover, the predicted stress fields and fracture patterns for the critical outer and inner wedge match remarkably well with the geometries and fracture modes inferred from the mineral veins in our study area (Figs 2 and 4; Supplementary Fig. 5).

## Discussion

**Figure 5:**
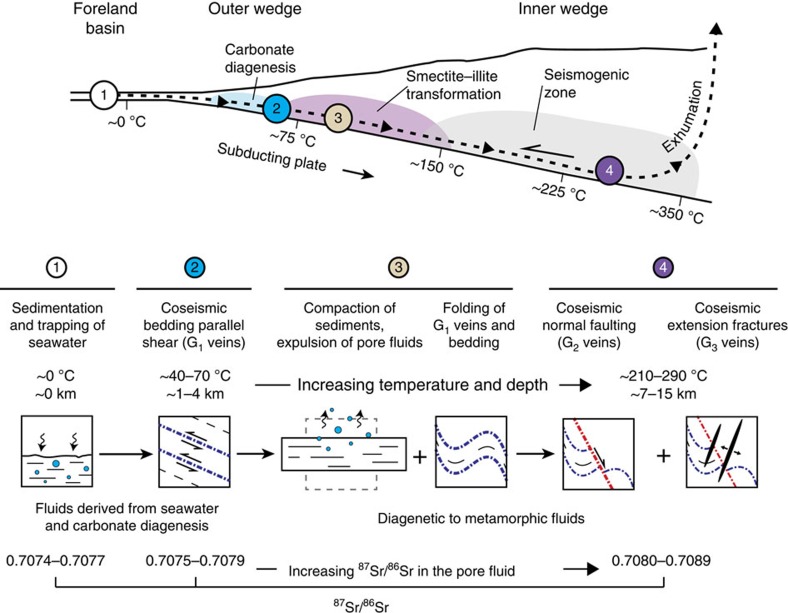
Different stages of vein formation along a particle path. (1) New sediments enter the subduction zone and are transported towards greater depth. (2) Coseismic compression of the outer wedge results in the formation of G_1_ veins. (3) The sediments are compacted and folded, pore water is expelled and remaining pore fluids are mixed with diagenetic-metamorphic fluids. (4) Coseismic extension within the inner wedge leads to the formation of G_2_- and G_3_ veins.

We have investigated the formation of mineral veins during the structural evolution of an accretionary complex, covering the range from initial frontal accretion to deep burial and low-grade metamorphism of the rocks (Fig. 5). By combining field data with geochemical and mechanical constraints, we learn that vein formation in the Alpine prism was restricted to distinct structural positions within the wedge. Vein formation in the outer wedge coincided with carbonate diagenesis at 1–4 km depth and at temperatures of ∼40–70 °C, that is, the same interval at which sediments experience the highest consolidation rates^39^. The combined effects of compaction and calcite cementation are likely to lead to a pronounced decrease in porosity, which promotes elevated pore fluid pressures in the accreted units. We suppose that, under these conditions the overpressured and mechanically weak rocks fail during coseismic compression of the outer wedge, resulting in the formation of G_1_ veins, as predicted by the Mohr–Coulomb wedge analysis.

In contrast, the formation of G_2_- and G_3_ veins took place in the inner wedge at elevated temperatures of ∼210–290 °C. Interestingly, the smectite–illite transformation and associated K-feldspar consumption^40^, which commonly occur at temperatures of 60–150 °C (ref. 41), must have happened before the formation of these veins. We suppose that the release of radiogenic ^87^Sr from clays and feldspars caused a first increase in the ^87^Sr/^86^Sr signature of the pore fluid, as recorded in the mineral veins. Moreover, the release of silica and subsequent quartz cementation is thought to facilitate the change from a velocity-strengthening to a velocity-weakening behaviour of the rocks, which is necessary for the onset of seismogenesis^42^. A striking feature of G_2_- and G_3_ veins is that they record stages of extensional faulting in an overall convergent setting, which has also been documented for other active and fossil accretionary complexes. For example, submarine structural mapping of the Nankai accretionary prism revealed the formation of normal faults along the crest of anticlines in the outer wedge^43^. Stratal extension and normal faulting have also been found along thrust faults in the Makran accretionary prism^44^. The occurrence of extension fractures in accretionary mélanges further suggests the persistence of tensile stress conditions during tectonic mélange formation^45^,^46^. Tension gashes in the palaeo-accretionary wedge of the South-Central Chilean forearc indicate extensional fracturing during exhumation of previously underplated rock units^47^. The latter case indicates that accretionary prisms may undergo prolongated stages of regional extension, which could have produced the observed superposition of G_2_- and G_3_ veins in the Alpine wedge, albeit without the envisioned dynamic effects outlined above. However, the extensional structures described in our study are neither associated with other large-scale tectonic structures indicative of an extensional stage within the Alpine wedge, nor are they related to specific deformation processes such as folding, thrusting or mélange formation. Moreover, the G_2_- and G_3_ veins developed on the prograde path and record a prominent switch from reverse faulting at shallow depths to extensional faulting at greater depths and temperatures. We therefore suggest that vein formation in the Alpine wedge illustrates the response of the wedge to megathrust earthquakes on the plate interface, which repeatedly shifted the wedge into a critical state. In this model, bedding-parallel shear veins (G_1_) originate from coseismic compression of the outer wedge, whereas normal faults and steep extension fractures (G_2_ and G_3_) were formed during short periods of extension within the inner wedge (Figs 4 and 5). For the latter, the strength of the basal detachment has to decrease coseismically from 0.04 to 0.01–0.02. This decrease is equivalent to stress drops of 50–75% and may suggest the need of great megathrust earthquakes. Average stress drops, however, have been found to be constant and independent of earthquake magnitude and commonly range between 1 and 10 MPa, with a mean value of around 3 MPa^48^. The shear stress on the megathrust is given by the friction law *τ*^F^=*μ*′_b_*σ*_n_, where *σ*_n_ is the normal stress and approximately the weight of the overburden^33^,^49^. At 11 km depth (that is, the average depth for the formation of G_2_- and G_3_ veins) *σ*_n_ is about 270 MPa and *τ*^F^∼11 MPa during the interseismic period. A coseismic decrease of *μ*′_b_ to 0.01–0.02 corresponds to stress drops of 5–8 MPa, indicating that the apparently high stress drops fall within the range of average values. Moreover, stress drops can vary significantly along the fault surface. While some patches experience complete stress drops others may even undergo stress increases^50^,^51^.

We conclude that faulting and vein formation in accretionary complexes can be triggered by megathrust earthquakes, which shift the wedge into an unstable state. This implies a coseismic increase in fracture permeability within the hanging wall of megathrusts, followed by a stable phase of interseismic fault sealing. Our work helps to understand how fractures are generated throughout the subduction earthquake cycle, which is essential to better-constrain the nature of postseismic fluid flow^52^ and to assess the seismic hazard of hydraulically driven aftershocks^53^.

## Methods

### Field investigations

The study area is located in the UNESCO World Heritage Swiss Tectonic Arena Sardona, in the northeastern part of the central European Alps (Supplementary Fig. 1). Structural elements (faults, mineral veins, bedding, cleavage and folds) were investigated along a ∼30-km-long northwest–southeast transect, comprising all three IFUs (that is, the central to southern part of the cross-section in Supplementary Fig. 1b). To approximate the orientation of structures at the time of formation, all structural measurements were corrected for exhumation-related block rotation, as constrained by apatite fission-track data^18^. The trend of the rotation axis is 70° (orogen-parallel); the magnitude of rotation is 10° (Supplementary Fig. 1c). We note that this correction does not affect the results or interpretations presented in this study. All samples for geochemical analyses were taken from two areas within the Globotruncana marl in the southern part of the study (marked by A1 and A2 in Supplementary Fig. 1a).

### Geothermal gradient

The modern average surface heat flow *Q*s in the study area is ∼80 mW m^−2^ (ref. 54). For the upper continental crust the temperature at a given depth can be calculated by (for example, see ref. 55)


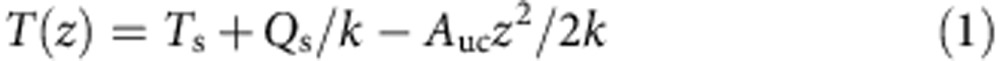


where *T*_s_ is the surface temperature (273 K), *k* the thermal conductivity (2.5 Wm^−1^ K^−1^) and *A*_uc_ the radiogenic heat production rate within the upper crust. Following Pollack and Chapman^56^, we assume that *A*_uc_ in the upper crust accounts for 40% of the surface heat flow, that is, *A*_uc_=0.4*Q*_s_/*D*_uc_, where *D*_uc_ is the thickness of the upper crust (taken to be 20 km). Solving equation (1) gives an average geothermal gradient of ∼27 °C km^−1^ for the upper 15 km of continental crust. As the surface heat flow may have been lower before the onset of continental collision and associated thickening of the continental crust, we assume a conservative range for the geothermal gradient in our study area of 20–30 °C km^−1^.

### Dynamic Mohr–Coulomb wedge analysis

The dynamic Mohr–Coulomb wedge theory provides exact stress solutions for an elastic-perfectly Coulomb plastic rheology and considers temporal variations of stresses within the wedge and along the basal detachment in subduction earthquake cycles^33^. We will briefly summarize the general concept of the theory and explain the main parameters constraining the model and refer the reader to refs 33, 49, 57 for further reading. The Coulomb wedge theory postulates that, the geometry of a wedge, which is given by the upper slope angle *α* and the basal dip *β*, is related to the strength of the wedge and the strength of the basal detachment^33^,^49^. The strength of the wedge is defined by the coefficient of internal friction *μ*, the cohesion gradient *η* and the pore fluid pressure ratio *λ*. The strength of the basal detachment is given by the effective coefficient of basal friction *μ′*_b_=*μ*_*b*_(1–*λ*_*b*_), where *μ*_b_ and *λ*_b_ denote the coefficient of basal friction and the basal pore fluid pressure ratio, respectively. The model is divided into an outer and an inner wedge, to account for differences in wedge strength and in seismogenic behaviour of the basal detachment. The outer wedge represents the near-trench part of accretionary wedges, which overlies the updip velocity-strengthening (aseismic) segment of the basal detachment and comprises young and weak sediments. The landward inner wedge overlies the velocity-weakening (seismogenic) part of the detachment and consists of well-consolidated older accreted units^33^. The subduction earthquake cycle is modelled by varying the effective coefficient of basal friction *μ′*_b_. For the interseismic period *μ′*_b_ is set to a fixed reference value, which is equal for both parts of the basal detachment. Megathrust earthquakes are considered by increasing and decreasing *μ′*_b_ along the aseismic and seismogenic part of the detachment fault, respectively. Given the strength and geometry of the wedge, the model calculates the effective stress ratio *m* for every value of *μ′*_b_, determining whether the wedge is stable (elastic deformation) or critical (at Coulomb failure). The results are pictured in a stability field diagram (Fig. 4; Supplementary Fig. 4), illustrating the critical values of basal friction *μ′*_b_ as a function of pore fluid pressure ratio *λ*. In addition, the model computes the maximum and minimum principal stresses (*σ*_1_ and *σ*_3_), as well as the angle of *σ*_1_ with the wedge surface *Ψ* as a function of basal friction *μ′*_b_ (Supplementary Fig. 5b).

All model parameters for the interseismic reference state are listed in Supplementary Table 4. The geometry of the Alpine accretionary wedge was constrained from geological cross-sections, which are based on surface information, borehole data and p-wave tomography models of the deep structure of the European Alps^35^. The errors on *α* and *β* due to uncertainties in the construction of cross-sections and velocity to depth conversion are estimated to be ±1° and ±2°, respectively^57^. The *μ′*_b_ value for the interseismic period was set to 0.04, according to values reported for the basal detachment in the central European Alps^34^, as well as for the Nankai and Cascadia subduction zones^33^,^58^. Following the above discussion, we defined the outer wedge to be weaker (*μ*=0.4, *η*=0.3) than the inner wedge (*μ*=0.7, *η*=0.6). The reference pore fluid pressure ratios for the outer and inner wedges were set to intermediate pore fluid pressures (*λ*=0.8) and high pore fluid pressures (*λ*=0.9), respectively (Supplementary Table 4).

We verified the validity of our results by testing the effect of different model parameters (Supplementary Fig. 4). For example, the transition from the outer to the inner wedge is often associated with a break in slope^33^,^57^, which cannot be assessed for exhumed palaeo-accretionary wedges. To take this into account, we examined the effects of a steeper outer and a flatter inner wedge and found no restrictions to the principle findings presented in this study (Supplementary Fig. 4a,d). The same applies to the other parameters (Supplementary Fig. 4). Finally, we constrained the precise conditions for normal faulting and extensional fracturing within the inner wedge (Supplementary Fig. 5). We calculated the differential stress and the orientation of *σ*_1_ for the inner wedge on the verge of failure (Supplementary Fig. 5a). This stress information was used to compute brittle failure-mode diagrams^59^,^60^, which illustrate the conditions of extensional fracturing and normal faulting (Supplementary Fig. 5b). We found that extensional fracturing is favoured at *λ*>0.95, whereas normal faulting occurs at *λ*<0.95, preferentially along pre-existing planes of weakness.

### Sample preparation

Thin sections of mineral veins and host rocks were analysed under a petrographic microscope. Mineral veins showing recrystallization or disequilibrium between quartz and calcite were excluded from geochemical analyses (Supplementary Fig. 2). From all other veins small blocks were cut out, crushed, washed and sieved. The 125–250-μm grain fraction was immersed in ethanol and 100–200 mg of inclusion-free quartz and calcite grains were hand-picked under a stereo microscope. Subsequently, the aliquots were washed in Milli-Q water and cleaned in an ultrasonic bath. Samples of unweathered host rock were cut into blocks, crushed and 10–15 g per sample were ground and homogenized in a tungsten-carbide mill.

### Stable isotopes and temperature estimates

δ^18^O values of quartz from G_2_- and G_3_ veins were obtained using a laser fluorination method^61^,^62^ and a Finnigan MAT 253 isotope ratio mass spectrometer at the University of Lausanne. Quartz samples were decarbonated in 1 M HCl for 4 h. Between 0.5 and 2 mg of sample material was loaded onto a Pt-sample holder and pumped out to a vacuum of about 10^–6^ mbar. After preflourination of the sample chamber, the samples were heated with a CO_2_-laser in 50 mbar of pure F_2_. Liberated O_2_ was purified through an extraction line and passing the gas over heated KCl salt (150 °C). The extracted O_2_ was collected on a molecular sieve (0.5 nm) and subsequently expanded into the mass spectrometer. Replicate oxygen isotope analyses of the standards used during the runs (*n*=19) yielded an external reproducibility of ≤0.20‰.

δ^13^C and δ^18^O values of vein and host rock calcites were measured at the University of Bern with a GasBench II connected to a Finnigan MAT Delta Plus XL mass spectrometer, using a He-carrier gas system^63^. About 250–1,000 μg of sample material was dissolved in concentrated H_3_PO_4_. All results are normalized using an in-house standard calibrated against δ^13^C and δ^18^O values of NBS-19 (1.95 and –2.20‰ VPDB, respectively). The external reproducibility for the analyses estimated from replicate analyses of the in-house standard (*n*=16) is ±0.05‰ for δ^13^C and ±0.04‰ for δ^18^O.

The temperature-dependent O isotope fractionation between quartz and calcite was calculated using empirically derived fractionation equations for Qz-H_2_O and Cc-H_2_O, which were determined for a temperature range of 200–500 °C and 0–500 °C, respectively^31^,^32^. Kim and O'Neil^64^ reported small differences in the oxygen isotope fractionations between calcite and water at temperatures <25 °C, which do not affect our calculations and are not further considered here. The 1*σ* error on the vein formation temperature was calculated by assuming a total 1*σ* error of ±0.24 in the δ^18^O measurements, that is, the sum of the external reproducibilities (±0.20‰ for δ^18^O_Qz_ and ±0.04‰ for δ^18^O_Cc_).

### Radiogenic isotopes

Rb–Sr isotopes were obtained for three samples of Globutruncana marl, comprising two samples with 50–60% calcite and one sample of shale containing no calcite. Aliquots of the two calcite-rich samples were treated with 1 M HCl for 5 min and subsequently centrifuged for 10 min at 3,000 r.p.m. to gain the solution and the residual fraction. Residuals, solutions and powdered bulk rocks were spiked with a mixed ^87^Rb–^84^Sr spike and dissolved in concentrated HF-HNO_3_. Insoluble residuals of organic matter were oxidized with concentrated H_2_O_2_.

^87^Sr/^86^Sr was measured for calcite from mineral veins and for bulk carbonate leachates of pure limestone. The bulk carbonate leachates were obtained by submerging small rock chips for 30 s in 0.1 M HNO_3_. The solutions were centrifuged for 10 min at 3,000 r.p.m. The vials were checked under the stereo microscope for eventual residuals, but no particles could be found.

Sr isotope measurements were performed using a TRITON Plus TIMS (Thermo-Fisher) at the mass spectrometer facility of the Institute of Geological Sciences at the University of Bern. The samples were loaded on rhenium ribbon single filaments in combination with a Ta_2_O_5_ activator. The measurement commenced when a signal intensity of 5 V on mass 88 was achieved. The ^87^Sr/^86^Sr ratios of the samples were normalized to a ^86^Sr/^88^Sr ratio of 0.1194 (ref. 65) using the exponential fractionation law. Samples were also corrected for the offset between the measured ^87^Sr/^86^Sr value of SRM987 of the individual session and the ^87^Sr/^86^Sr ratio of 0.71024 (ref. 26). The external reproducibility (2 s.d.) of our method is ±0.00005.

Rb isotopes were measured on a Nu Instruments inductively coupled plasma-source multicollector mass spectrometer at the mass spectrometer facility of the Institute of Geological Sciences at the University of Bern. The samples were measured using bracketing standards for each sample and corrected for the offset between the measured ^87^Rb/^85^Rb value of the in-house standard and its nominal value (^87^Rb/^85^Rb=0.38581). The external reproducibility (2 s.d.) estimated from the replicate analyses of the standard (*n*=8) is ±0.00012. The age-dependent ^87^Sr/^86^Sr ratios of the three host rock samples given in the text and in Fig. 3 are recalculated to the time of metamorphism (25 Ma, ref. 66), using the ^87^Rb decay constant of 1.42 × 10^−11^ a^−1^ (ref. 67).

## Additional information

**How to cite this article:** Dielforder, A. *et al.* Linking megathrust earthquakes to brittle deformation in a fossil accretionary complex. *Nat. Commun.* 6:7504 doi: 10.1038/ncomms8504 (2015).

## Supplementary Material

Supplementary Information Supplementary Figures 1-5, Supplementary Tables 1-4 and Supplementary References
