# Supplementary material for: Linking megathrust earthquakes to brittle deformation in a fossil accretionary complex
Source: Nat Commun. 2015 Jun 24;6:7504. doi: 10.1038/ncomms8504 (PMC4491836; doi:10.1038/ncomms8504)
Supplement: Supplementary Information — Supplementary Figures 1-5, Supplementary Tables 1-4 and Supplementary References [file ncomms8504-s1.pdf]

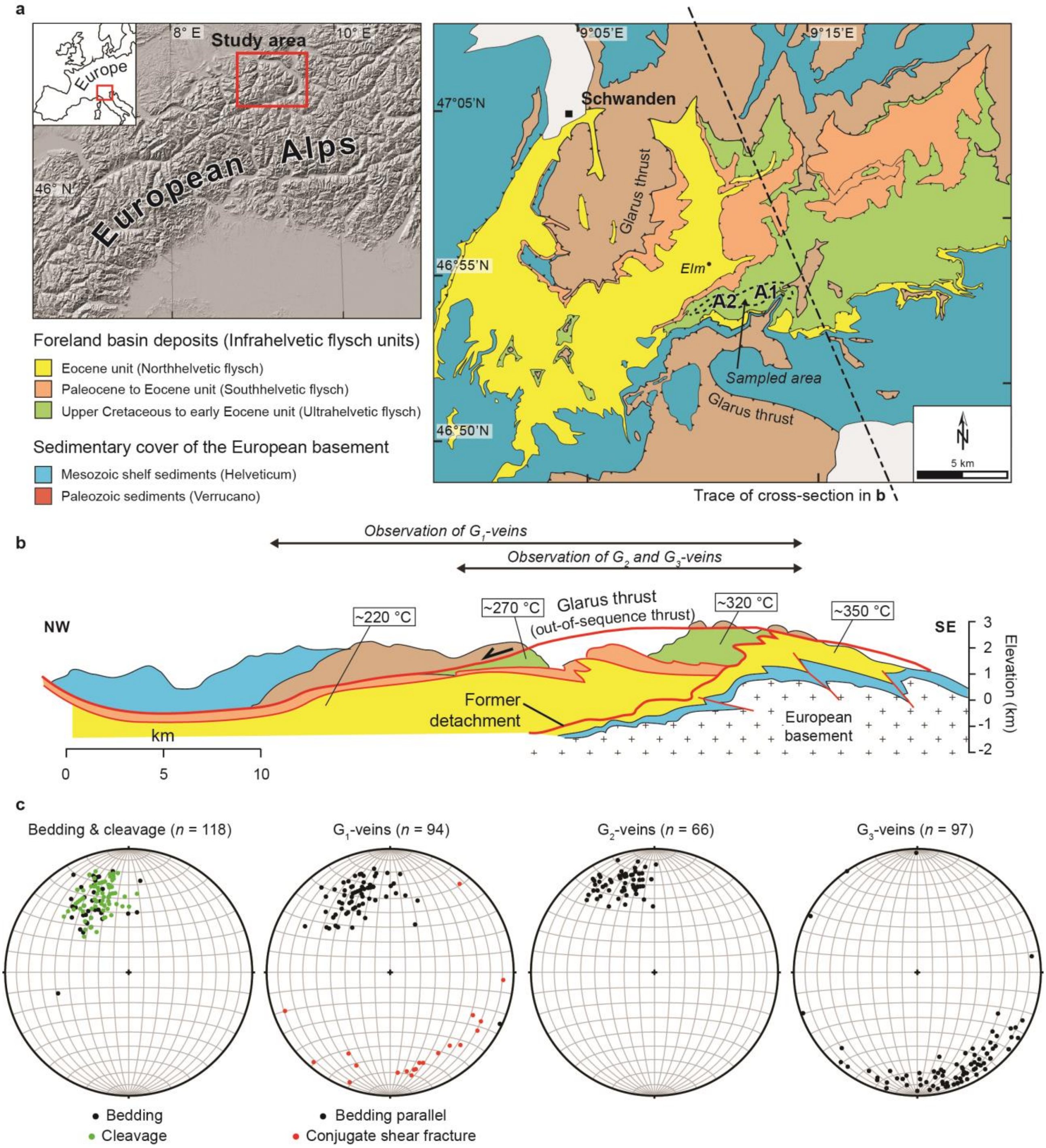

Supplementary Figure 1 | Geological setting of the study area. a, Geographic location and tectonic map of the study area[1]. The sedimentary cover of the European basement was thrusted along the Glarus fault on top of the Infrahelvetic flysch units during the late stage evolution of the accretionary complexes. The dashed line indicate the Globotruncana marl within the Ultrahelvetic thrust slice. A1 and A2 mark the areas from which samples for geochemical analyses were taken. **b,** Schematic geological cross-section[1,2] illustrating out-of-sequence-thrusting along the Glarus thrust (cf. Fig. 1). The study area is located in the central and southern part of the cross-section. The arrows on top indicate the range of observation for G_1-veins as well as G_2- and G_3-veins. **c**, Stereoplots showing poles to bedding, cleavage, G_1-, G_2-, and G_3-veins. Bedding and cleavage are subparallel. The majority of G_1-veins is parallel to bedding. A minor set of conjugate shear fractures were formed at an angle of ~60 ° to bedding parallel veins. Both orientations record reverse faulting in the study area. G_2-veins are preferentially aligned along bedding/cleavage planes similar to G_1-veins, but were formed at a later stage and indicate normal faulting in the study area. G_3-extension veins dip steeply to the north and record a similar stress field during vein formation as G_2-veins.

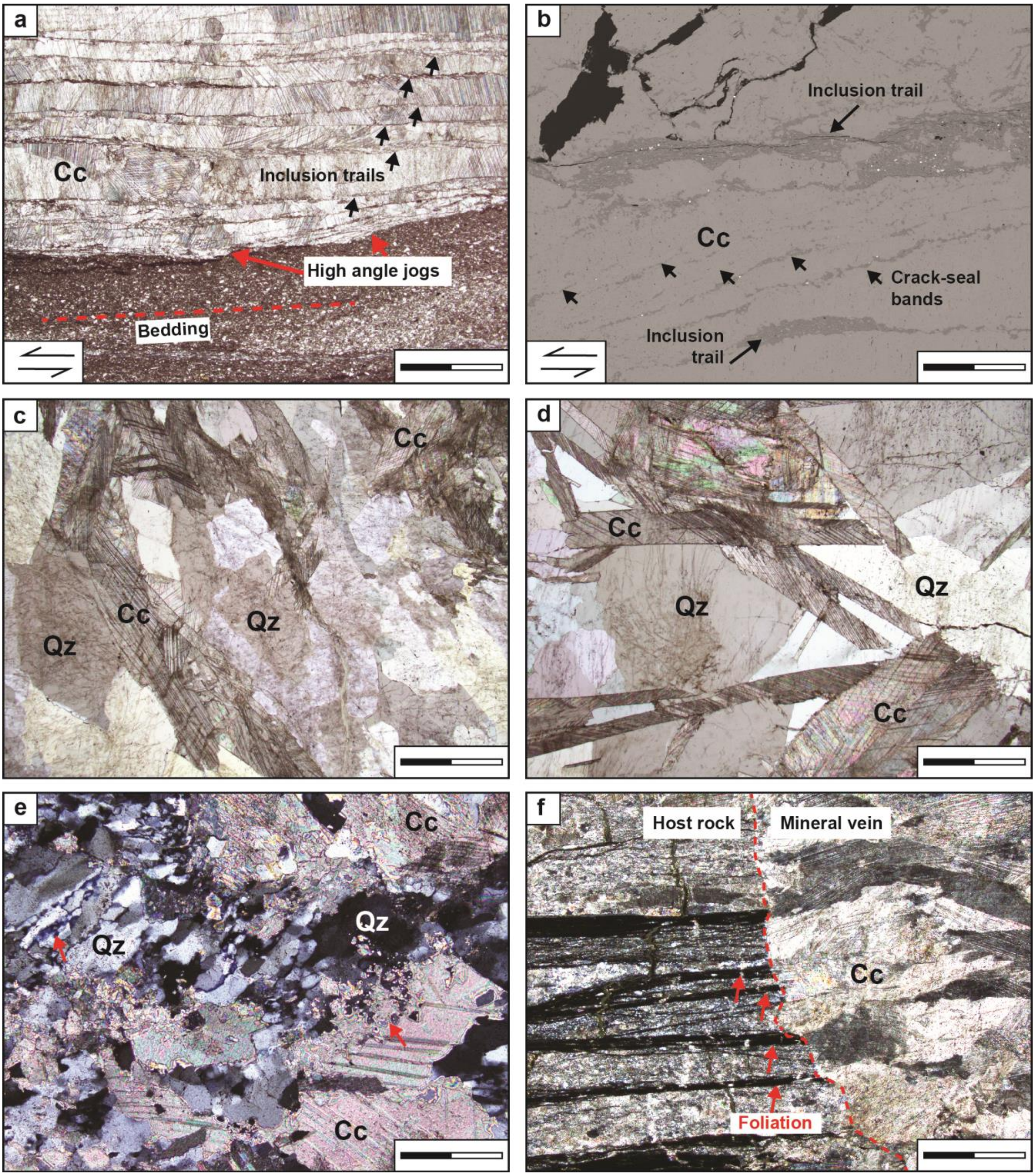

Supplementary Figure 2 | Example of mineral veins. Photographs taken under a petrographic microscope (**a, c-f**) and a scanning electron microscope (**b**). Cc = calcite, Qz = quartz. **a,** Bedding parallel G_1-vein. The red arrows indicate high angle jogs, the black arrows inclusion trails. Shear sense is sinistral. Further information on the microstructures of these veins can be found in ref. 3. Plane polarized light. Scale bar 1 mm. **b**, Backscattered-electrons image of a bedding parallel G_1-vein. The inclusion trail consist of sheared host rock fragments. The crack-seal bands trace the successive formation of the vein and comprise quartz and some small particles of host rock. Shear sense is sinistral. Scale bar 200 µm. **c,d,** Examples of quartz and calcite crystals in a G_2- and G_3-vein, respectively. The microstructural texture suggests that both phases grew simultaneously in equilibrium with the pore fluid. Crossed polars. Scale bar in (**c)** 2 mm and 1.5 mm in (**d). e**, Quartz and calcite crystals in a G_3-vein, which show no equilibrium between the phases and are affected by incipient recrystallisation (red arrows). Such samples were excluded from geochemical analysis. Crossed polars. Scale bar 200 µm. **f**, G_3-vein cross cutting foliation (red arrows). Crossed polars. Scale bar 750 µm.

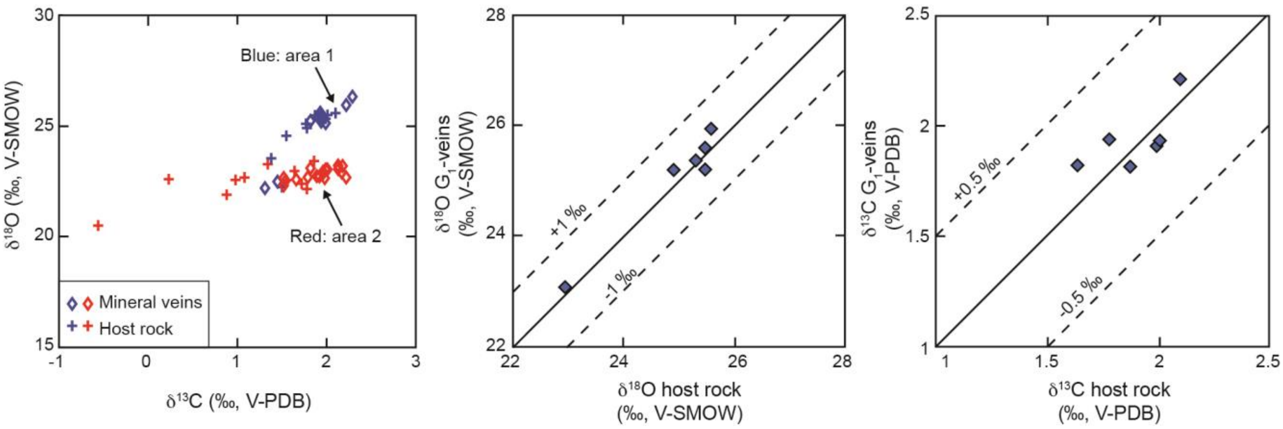

Supplementary Figure 3 | Stable isotopes systematics. a, δ^{18}O and δ^{13}C values of calcite cements from host rock samples (crosses) and calcite from mineral veins (diamonds). The samples were taken from two different areas within the Globotruncana marl (Supplementary Fig. 1a). The values of mineral veins generally overlap with the respective values of host rock, indicating a rock-buffered fluid during vein formation. The distinct trends for area 1 and area 2 reflect slight temperature differences in the onset of carbonate diagenesis, and respective δ^{18}O values of calcite cements. **b, c**, Detail of (**a**). The paired δ^{18}O and δ^{13}C values for calcite from G_1-veins and calcite cements from adjacent host rock samples plot along a 1:1 line.

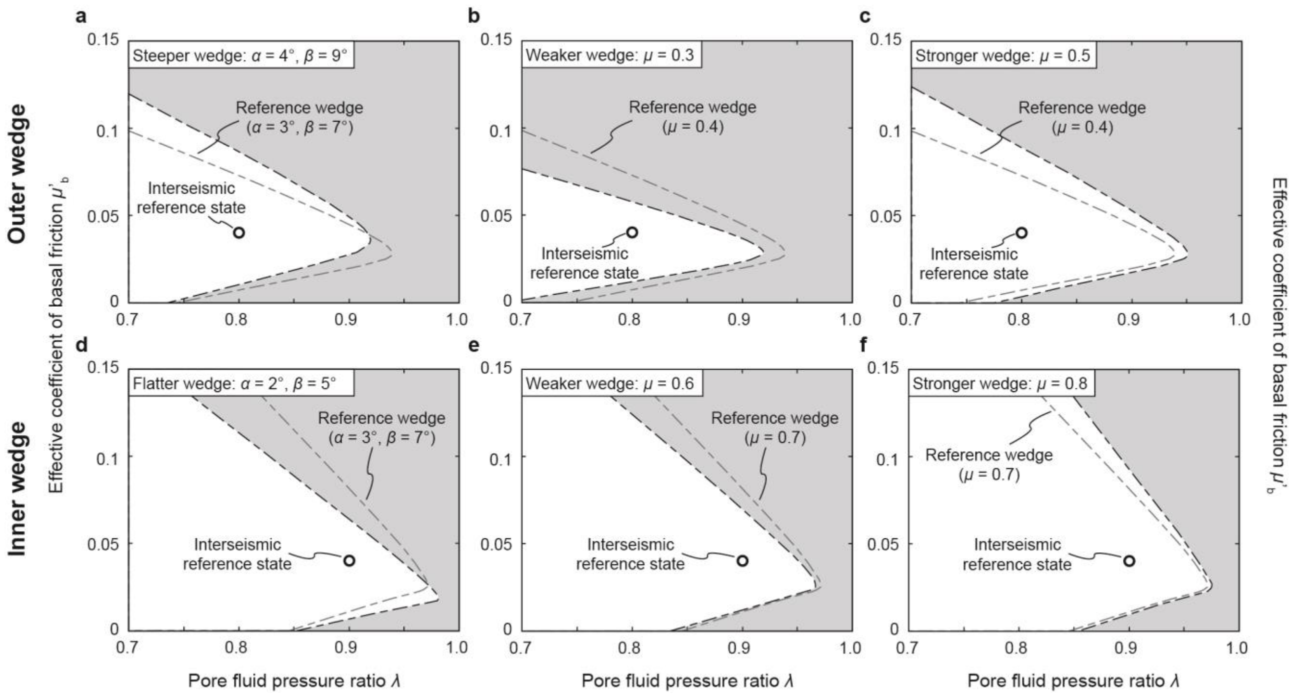

Supplementary Figure 4 | Sensitivity analysis. Stability field diagrams illustrating the effect of model parameters on the stability of outer and inner wedges. The grey dashed lines indicate the reference model, the white area highlights the stability range of the respective model run **a, d,** Effects of a steeper and flatter wedge geometry on the outer and inner wedge, respectively. **b, e,** Effects of a weaker wedge material due to a smaller coefficient of internal friction. **c, f,** Effects of a stronger wedge material due to a greater coefficient of internal friction.

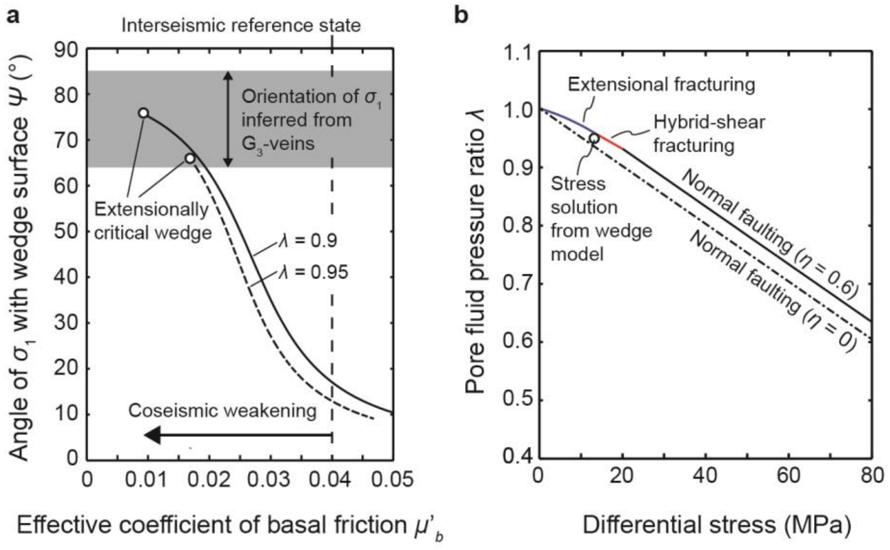

Supplementary Figure 5 | Stress and failure conditions for the inner wedge. a, Elastic stress paths for the inner wedge illustrating the angle of σ_1 with the wedge surface Ψ as a function of the effective coefficient of basal friction μ'_b. Two exemplary solutions for pore fluid pressure ratios of λ = 0.9 and λ = 0.95 are given. During megathrust earthquakes μ'_b decreases and Ψ becomes greater. The wedge remains stable along the whole stress paths. At sufficiently high stress drops the wedge becomes extensionally critical as marked by the open circles. The modeled orientations of σ_1 at wedge failure overlap with the orientation of σ_1 inferred from G_3-veins from our study area (grey area). **b,** Brittle failure mode diagram illustrating the conditions for extensional fracturing, hybrid-shear fracturing, and normal faulting in the inner wedge. Two solutions for normal faulting are shown: normal faulting in cohesive rocks (η = 0.6) and normal faulting in cohesionless rocks or along preexisting planes of weakness (η = 0). The open circle indicates an exemplary stress solution for the inner wedge on the verge to failure. At a pore fluid pressures ratio λ < 0.95 the wedge is likely to fail by normal faulting along a preexisting plane of weakness (e.g. foliation), whereas at λ > 0.95 extensional fracturing is favoured.

Supplementary Table 1 | Stable isotopes and $^{87}Sr/^{86}Sr$ ratios of mineral veins and bulk carbonate leachates

Sample ID	Sample area[a]	$\delta^{13}C_{Cc}$[b] (‰)	$\delta^{18}O_{Cc}$[c] (‰)	$\delta^{18}O_{Qz}$[c] (‰)	$\Delta^{18}O_{Qz-Cc}$ (‰)	Temperature (°C) ± 1σ[d]	$^{87}Sr/^{86}Sr$[e]
G1, calcite shear veins							
13F76	A1	□□□□	□□□□□	□	□	-	0.70763
13F76-CSF	A1	1.94	25.43	-	-	-	0.70757
13F79-A	A1	2.22	25.93	-	-	-	0.70787
13F79-B	A1	2.29	26.31	-	-	-	0.70757
13F80-A	A1	1.93	25.59	-	-	-	0.70776
13F80-B	A1	-	-	-	-	-	0.70779
13F80-CSF	A1	1.94	25.28	-	-	-	0.70771
13F81-A	A1	1.94	25.18	-	-	-	0.70774
13F81-B	A1	-	-	-	-	-	0.70770
13F81-CSF	A1	1.99	25.11	-	-	-	0.70750
13F83	A1	1.82	25.21	-	-	-	0.70770
14F42	A2	1.82	23.08	-	-	-	0.70789
14F43	A2	1.52	22.64	-	-	-	0.70790
G2, quartz-calcite normal fault cores							
13F64-A	A2	1.96	22.84	-	-	-	0.70818
13F64-B	A2	1.92	22.71	-	-	-	0.70830
13F77	A1	1.45	22.47	24.58	2.11	259 +33/-28	0.70862
14F32	A2	-	-	-	-	-	0.70832
14F33	A2	-	-	-	-	-	0.70828
14F37	A2	2.17	22.89	25.31	2.42	224 +27/-23	0.70820
14F38	A2	2.13	23.20	25.41	2.21	247 +31/-26	0.70808
14F39	A2	-	-	-	-	-	0.70810
14F48	A2	2.01	23.02	25.54	2.52	214 +25/-22	0.70804
14F49	A2	1.52	22.30	24.39	2.09	262 +33/-28	0.70858
14F50	A2	2.18	23.18	25.28	2.10	260 +33/-28	0.70836
14F55	A2	1.89	22.72	25.19	2.47	219 +26/-22	0.70822
G3, quartz-calcite extension fractures							
13F44	A2	1.54	22.51	24.47	1.96	279 +37/-31	0.70886
13F61	A2	1.79	22.67	25.16	2.49	217 +25/-22	0.70836
13F62	A2	1.96	22.92	-	-	-	0.70824
13F89	A1	1.31	22.18	24.08	1.90	287 +39/-32	0.70880
14F41	A2	-	-	-	-	-	0.70823
14F45	A2	2.13	23.04	25.52	2.48	218 +26/-22	0.70808
14F47	A2	1.99	23.02	-	-	-	0.70813
14F31	A2	-	-	-	-	-	0.70838
14F30	A2	-	-	-	-	-	0.70837
14F51	A2	1.98	22.63	25.11	2.48	218 +26/-22	0.70830
14F52	A2	1.66	22.57	24.85	2.28	239 +29/-25	0.70844
14F53	A2	2.22	22.66	25.02	2.36	230 +28/-24	0.70830
Bulk carbonate leachates from limestone sample 13F79, 4 measurements (M1-M4)							
13F79-M1	A1	2.10	25.57	-	-	-	0.70769
13F79-M2	A1	-	-	-	-	-	0.70767
13F79-M3	A1	-	-	-	-	-	0.70770
13F79-M4	A1	-	-	-	-	-	0.70768

[a] The sample areas A1 and A2 are situated within the Globotruncana marl and are indicated in Supplementary Fig. 1

[b] $\delta^{13}C$ values are given relative to the V-PDB standard. The 1σ error on the $\delta^{13}C_{Cc}$ measurements is given by the external reproducibility of ± 0.05 ‰.

[c] $\delta^{18}O$ values are given relative to the V-SMOW standard. The 1σ errors on the $\delta^{18}O_{Cc}$ and $\delta^{18}O_{Qz}$ measurements are given by the external reproducibilities of ± 0.04 ‰ and ± 0.2 ‰.

[d] The 1σ errors on vein formation temperatures are calculated by assuming a total 1σ error of ± 0.24 ‰ in the $\delta^{18}O$ measurements.

[e] The 2σ error on the $^{87}Sr/^{86}Sr$ ratios is given by the external reproducibility of our method and is ± 0.00005.

Supplementary Table 2 | Stable isotopes of calcite cements

Sample ID[a]	$\delta^{13}C_{Cc}$ (V-PDB)[b] (‰)	$\delta^{18}O_{Cc}$ (V-SMOW)[b] (‰)
Sample area A1		
13F74-HR	1.38	23.52
13F75-HR	1.77	25.08
13F76-HR	1.99	25.31
13F77-HR	1.50	22.22
13F79-HR	2.10	25.57
13F80-HR	2.01	25.48
13F81-HR	1.55	24.54
13F82-HR	1.78	24.89
13F86-HR	1.87	25.46
Sample area A2		
13F30-HR	0.88	21.88
13F31-HR	1.55	22.54
13F39-HR	1.86	23.40
13F38-HR	0.23	22.58
13F42-HR	0.98	22.55
13F43-HR	1.08	22.66
13F47-HR	1.78	22.14
13F56-HR	-0.56	20.49
14F30-HR	1.72	22.38
14F42-HR	1.64	22.95
14F43-HR	1.34	23.26

[a] The sample areas A1 and A2 are situated in the Globotruncana marl and are indicated in Supplementary Fig. 1.
[b] The 1σ errors on the $\delta^{13}C_{Cc}$ and $\delta^{18}O_{Cc}$ measurements are given by the external reproducibilities of ± 0.05 ‰ and ± 0.04 ‰, respectively.

Supplementary Table 3 | Radiogenic isotope data of host rock samples

Sample ID[a]	Rb (ppm)	Sr (ppm)	$^{87}Rb/^{86}Sr$	$^{87}Sr/^{86}Sr$	$(^{87}Sr/^{86}Sr)i$ = 25 Ma[b]	$(^{87}Sr/^{86}Sr)i$ = 46 Ma[b]
13F35-HR	202	369	1.581 ± 0.002	0.71028 ± 0.00004	0.70971	0.70923
13F43-HR	56	398	0.4106 ± 0.0006	0.70914 ± 0.00002	0.70899	0.70887
13F43-R	106	23	13.61 ± 0.14	0.72233 ± 0.00002	0.71743	0.71330
13F43-L	216	1071	0.58 ± 0.37	0.70874 ± 0.00002	0.70853	0.70835
13F47-HR	56	764	0.213 ± 0.002	0.70887 ± 0.00001	0.70879	0.70873
13F47-R	93	10	26.1 ± 0.1	0.73010 ± 0.00002	0.72069	0.71278
13F47-L	109	1135	0.27 ± 0.02	0.70850 ± 0.00001	0.70840	0.70832

[a] HR = host rock, R = residual (insoluble in 0.1 M HNO_3), L = leachate (soluble in 0.1 M HNO_3).
[b] $^{87}Sr/^{86}Sr$ ratios back calculated to the time of metamorphism[4] in the IFU (~25 Ma) and to initial frontal accretion[5] (~46 Ma).

Supplementary Table 4 | Model parameters

	Outer Wedge	Inner wedge
Upper slope angle (°)	3 ± 1	3 ± 1
Basal dip (°)	7 ± 2	7 ± 2
Coefficient of internal friction	0.4 ± 0.1	0.7 ± 0.1
Cohesion gradient	0.3	0.6
Pore fluid pressure ratio	0.8	0.9
Effective coefficient of basal friction	0.04	0.04
Sediment density (kg m-3)	2500	2600
Tensile strength (MPa)	not defined	3

Supplementary References

1. Pfiffner, O.A. et al. Structural map of the Helvetic zone of the Swiss Alps, including Voralberg (Austria) and Haute Savoie (France). Geological Special Map 128/1 – 7. *Swisstopo/Landesgeologie*, (2011).
2. Lihou, J.C. Structure and deformational history of the Infrahelvetic flysch units, Glarus Alps, eastern Switzerland. *Eclogae Geol. Helv.* **89**, 439–460 (1996).
3. Koehn, D. Passchier, C.W. Shear sense indicators in striped bedding-veins. *J. Struct. Geol.* **22**, 1141–1151 (2000).
4. Hunziker, J.C. *et al*. The evolution of illite to muscovite; mineralogical and isotopical data from the Glarus Alps, Switzerland. *Contrib. Mineral. Petr.* **92**, 157–180 (1986).
5. Pfiffner, O.A. Evolution of the north Alpine foreland basin in the central Alps. *Int. Assoc. Sediment. Spec. Publ.* **8**, 219–228 (1986).
